# Human olfactory consciousness and cognition: its unusual features may not result from unusual functions but from limited neocortical processing resources

**DOI:** 10.3389/fpsyg.2013.00819

**Published:** 2013-11-01

**Authors:** Richard J. Stevenson, Tuki Attuquayefio

**Affiliations:** Department of Psychology, Macquarie UniversitySydney, NSW, Australia

**Keywords:** olfaction, function, consciousness, comparative, cross-modal

## Abstract

Human and animal olfactory perception is shaped both by functional demands and by various environmental constraints seemingly peculiar to chemical stimuli. These demands and constraints may have generated a sensory system that is cognitively distinct from the major senses. In this article we identify these various functional demands and constraints, and examine whether they can be used to account for olfaction's unique cognitive features on a case-by-case basis. We then use this as grounds to argue that specific conscious processes do have functional value, a finding that naturally emerges when a comparative approach to consciousness across the senses is adopted. More generally, we conclude that certain peculiar features of olfactory cognition may owe more to limited neocortical processing resources, than they do to the challenges faced by perceiving chemical stimuli.

## Introduction

The aim of this manuscript is to explore two ideas. The first, and the one that occupies the most space, is that in human olfaction there are function-related reasons why consciousness and cognition are instantiated in an unusual way relative to the major senses (e.g., Herz and Engen, 1996; Zucco, 2003). To address this, we start by reviewing the main parameters that govern olfactory perception to identify and evaluate potential functional causes of olfaction's unusual features. This is followed by a case-by-case examination of the major differences in olfactory consciousness-related processing that have been identified in the literature. Included within this section are three broad types of finding: (1) differences in the content of consciousness (single vs. multiple representations; primacy of affect; universal synesthesia); (2) differences in attentional control and access (smell is a dual sense but without dual awareness; failure to reinstate the representation of a dishabituated smell); and (3) differences in conscious correlates of post-perceptual processing (“imagery” and “rehearsal” without conscious correlates). In each case we review the evidence for the claim of “specialness” and follow this with an examination of how it might relate to olfactory function. The final part of the manuscript examines the second idea, namely the value associated with taking a comparative, cross-modal approach to consciousness and cognition.

## The human olfactory system

The purpose of this section is to evaluate whether there are any unusual or unique aspects of human olfaction, with respect to stimulus and function that might explain its atypical psychological features. The olfactory system's principal function is to recognize the airborne (i.e., typically volatile) chemical correlates of biologically significant events (e.g., Wilson and Stevenson, 2006). In humans, these biologically significant events primarily relate to ingestion (e.g., detection of food), avoidance of environmental hazards (e.g., gas leaks) and social communication (e.g., kin recognition; Stevenson, 2010). The avoidance of environmental hazards, and social communication, all rely on recognizing the volatile chemical cues that are associated with these things—be it a predator or a potential mate. Section Chemicals as Stimuli (Chemicals as stimuli) considers whether the nature of the chemical stimulus places any particularly unusual burdens upon the brain.

While environmental hazards and social communication mainly rely upon the recognition of volatile chemicals in the external environment, ingestion-related olfaction brings with it a more unique problem. This is because the olfactory system has to associate events in the external world, namely the smell of food, with events in the body, such as the taste of food in the mouth and its delayed consequences—fullness, nausea etc. Section The Special Demands Imposed by Eating (The special demands imposed by eating) examines this particular problem.

### Chemicals as stimuli

An important consideration when examining olfaction is to understand the physical stimulus that the system has evolved to detect (Hudson, 1999). Most chemical correlates of biological events are complex mixtures composed of tens or hundreds of different chemical components (e.g., Maarse, 1991). These mixtures contain varying amounts of each chemical, with the higher concentration components probably dominating perception (Weiss et al., 2012). Nonetheless, multiple low concentration components, even when each is below its own detection threshold, can act together to generate a smell (Laska and Hudson, 1991). The brain then has to recognize such multi-component mixtures, and in addition it also has to deal with stimulus fidelity. Chemical mixtures degrade in the environment through the effects of sun, rain and wind. In addition, there are many variations in the chemical mixtures coming from any given class of emitter (e.g., prey odor may change with diet, age, gender, health status etc.). In all of these cases the brain still needs to be able to recognize a weakened or variant signal.

A further problem concerns the continued presence of the same chemicals in one particular area. These may continue to stimulate olfactory receptors potentially masking the detection of new chemical events. Finally, and perhaps most importantly of all, chemical stimuli are poorly placed to support a flexible communication system. A flexible communication system is one in which novel information (e.g., via combining existing signals) can be transmitted and received in contrast to a fixed system where particular signals communicate just one or a limited range of meanings. While use of fixed chemical signals is a widespread feature of many animal communication systems, including in mammals (e.g., Broad and Keverne, 2008), chemicals cannot be readily used to transmit information flexibly, in the way that light and vibration can. A failure to support flexible communication is perhaps one reason why olfaction has remained a relatively minor sense in humans and in other higher mammals. And it is a minor sense, even though it is a highly sensitive and discriminating one (Yeshurun and Sobel, 2010). One reason for making such a claim is that it is easier to live without smell (anosmia), than it is to live without audition or vision. This can be seen in compensation provisions made by the AMA's 1993 *Guide to the Evaluation of Permanent Impairment*, which regards loss of smell as a 3% impairment, relative to deafness at 35% and blindness at 85%. While these figures cannot accurately reflect the full loss associated with anosmia (e.g., Hummel and Nordin, 2005), they do reflect the general distinction in utility between the major senses and smell.

The biological system that has evolved to detect and recognize volatile chemicals shares many features in common with the systems used to detect visual and auditory stimuli (Wilson and Stevenson, 2006). Thus, excepting the flexible communication issue, the problems outlined in the preceding paragraphs for chemical stimuli are *conceptually* similar to those for electromagnetic or vibratory stimuli. Chemicals are detected in humans by around 40 million olfactory receptor neurons located on the olfactory epithelium (Cunningham et al., 1999). Each side of the nose has its own discrete olfactory epithelium, which is positioned inside the upper part of the nasal cavity lying mainly on the cribiform plate (Doty, 2001). Each olfactory receptor neuron expresses one out of a large range of different G-protein coupled receptor types (hereafter, odorant receptors; Buck and Axel, 1991). In humans, there appear to be around 413 different odorant receptors (Glusman et al., 2001; Olender et al., 2012). It is the odorant receptor type that dictates the form of ligand that binds to each olfactory receptor neuron.

Animal research suggests that odorant receptor types are quite broadly tuned, so while maximally responsive to a group of related chemicals, they will still fire to more distantly related ones, especially at higher concentrations (Malnic et al., 1999). This is an important observation, because when combined with the finding that receptor neurons expressing the same odorant receptor types converge to form structures called glomeruli (Ressler et al., 1993), it suggests the basis for a pattern recognition system. That is the spatial activation across the glomeruli, as well as changes in activation over time, provide the input to a content addressable recognition memory system located within primary olfactory cortex (Haberly, 2001). Not only can a content addressable memory system recognize complex patterns and learn new ones, it can also recognize weakened and variant inputs. The primary olfactory cortex has another feature, in that it stops responding to the same receptor input very quickly, allowing it to filter out background stimulation. This occurs even though there is relatively little alteration in peripheral receptor input as measured in rodents (Wilson and Linster, 2008). A similar picture is supported in humans, with neuroimaging suggesting that primary olfactory cortex rapidly adapts to continuous odorant stimulation (Sobel et al., 2000).

While having 400 or so different odorant receptor types is clearly quite unlike vision or audition, the solution adopted to recognizing discrete events in the chemical world is similar to recognizing visual or auditory objects, namely a memory based pattern recognition system (see Stevenson, 2013a). Olfaction then is a system robustly capable of recognizing (and learning) complex stimulus inputs (and weakened or varietal inputs), with constant adjustment via habituation to maintain sensitivity to change.

### The special demands imposed by eating

One of the most important functions of the olfactory system concerns food selection (Hoover, 2010). This is especially so for omnivores, who need to remember the nutritional value of many different foods, and to avoid eating those that have made them sick (Rozin, 1976). This requires the olfactory system to perform an unusual feat. *De novo*, information about the nutritional value of a potential food can only be obtained via oral sampling (i.e., taste and somatosensation) and digestion (i.e., from nutrient signals arising in the gut). If smell is to signal the nutritional value of a food, then the body needs some way of detecting the smell of food in the mouth (so that the correct odor is targeted), so that this olfactory signal can then become associated with the food's nutritional value. A food's smell alone can then come to signal its potential nutrient value without the need for oral sampling (e.g., Hiramatsu et al., 2009). For this reason the olfactory system is able to perceive volatile chemical signals arising externally *and* internally, in the latter case via a set of nostrils (nares) located at the back of the throat (Mozell et al., 1969).

Smelling via the posterior nares (nostrils) in the throat is termed retronasal olfaction, in contrast to sniffing through the nose or anterior nares, which is termed orthonasal olfaction. While retronasal chemical stimulation is detected and processed in largely the same manner as orthonasal stimulation, it is not normally accompanied by any conscious awareness that it is an “olfactory” input. Instead, lay people refer to the sensory experience of eating and drinking as “taste” or “flavor” (Rozin, 1982; noting that flavor is the preferred technical term). The olfactory signals arising from food in the mouth can come to be associated with both the food's immediate (is it sweet, bitter, burning etc in the mouth?) and delayed consequences (is it nutritious or poisonous?; see Brunstrom, 2004). This enables the olfactory system to provide, encapsulated within the smell percept, information about the nutritional correlates of a potential food (e.g., is it energy dense?) when it is later encountered in the external world and is being evaluated for consumption. This olfactory information is then used to make decisions about ingestion in both people and animals (e.g., Hiramatsu et al., 2009). No other sense has this dual system architecture.

### Conclusions about the human olfactory system

The argument advanced in this section is that while physiologically olfaction has some unique solutions to perceiving odors (e.g., 350 different receptor types), the fundamental conceptual basis of this process is similar to that of the major senses (see Wilson and Stevenson, 2006; Stevenson, 2013a). Nonetheless, two function-related differences emerge as potentially important. One, which is highly distal, concerns the inflexibility of chemical stimuli as a communication medium. This may have contributed to olfaction remaining a minor sense in humans and other higher mammals. The other, which is more proximal, concerns food. Food choice requires the linking of external and internal events and this may have introduced information processing approaches unique to olfaction.

## Differences between processing in olfaction and other modalities

In this section three types of processing differences are examined. The first concerns the content of olfactory consciousness, which is used here to refer to both the nature of that content and also its quantity (i.e., one or many percepts). This includes the unitary and serial nature of odor percepts, the dominance of affective processing and the presence of universal odor-induced taste synesthesia. The second concerns attention, and examines the dissociation between awareness of the content of consciousness and the sense modality generating that content, and the apparent absence of voluntary dishabituation. The third looks at the role of conscious post-perceptual processing in olfactory cognition (i.e., imagery and rehearsal). In each of these cases we start by outlining the evidence and then examine how the difference may relate to functional aspects of olfactory processing.

### Content of consciousness

#### Perceiving one smell at a time

In vision and audition, it has been argued that what can be experienced at any given moment exceeds what can be processed in greater detail. This is best captured experimentally in a series of studies reported by Sperling (1960). In a prototypical experiment, participants were briefly presented with a grid of letters, all of which they reported seeing. After the stimuli had vanished participants could only accurately recall a subset of the viewed set. Similar distinctions between the apparent phenomenal wealth of visual experience, contrasted with the more limited amount that can be accurately reported have now been observed in many studies (e.g., Simons and Chabris, 1999; Simons and Rensink, 2005). Block (2005, 2007) has described this succinctly as “phenomenal content overflowing accessibility” with somewhat similar distinctions being drawn by other authors (Dehaene and Naccache, 2001; Edelman, 2003). In olfaction, this does not seem to be the case. Olfaction might be better characterized as either “phenomenal content equals accessibility” or perhaps “there is only accessible content.”

Many researchers have described olfactory experience as unitary (e.g., Yeshurun and Sobel, 2010; but not all, see Auffarth, 2013). By this it seems they mean: (1) that an olfactory percept cannot be readily broken into a set of parts; and (2) that the whole has some sense of coherence. There is some support for this type of definition. That odors cannot be readily decomposed into their component parts is suggested by an extensive series of experiments reported by Laing and colleagues (e.g., Laing and Glemarec, 1987; Livermore and Laing, 1996). In these studies participants were trained to identify particular odors. These odors were then combined into mixtures of increasing complexity and participants' task was to identify which odor or odors were present. A consistent finding has been that participants and even industry experts (e.g., perfumists) cannot reliably detect more than three odors in a mixture. This would seem to set one possible upper limit on the content of olfactory consciousness.

This upper limit of around three may be unduly optimistic. First, in these studies all of the participants were pre-trained to identify the odors alone, suggesting that the default mode of processing may be to treat “a smell” as precisely that—a singular “one smell.” Second, the usual procedure adopted in these experiments is to ask participants to determine if a particular component is present in a mixture (i.e., a selective attention approach). What this procedure cannot tell us is whether it is possible to serially scan an odor mixture (or indeed to experience all three at once), and experience successively different percepts as each component is recognized in turn. We investigated this possibility, albeit indirectly, in a recent series of experiments using binary odor mixtures (Stevenson and Mahmut, 2013a). When participants experience a blend of two odors, there is evidence that across the course of successive presentations, perception can shift from that of a blend to perceiving mainly one component or the other. What is striking about these results is that participants cannot seemingly detect these transitions, even though evidence that they have occurred can be found in their ratings. Even alerting participants to the nature of the task does not improve awareness. These results suggest that when a stimulus is smelled it is perceived by default as *a* smell, and that even if it contains two or more detectable components, these may be hard to notice over successive exposures.

While detecting individual smells within an odor mixture may represent one type of multidimensionality, it may not be the only type. Odors can be characterized by their capacity to remind people of other smells, a phenomenon termed redolence (e.g., Dravnieks, 1982). How many they remind someone of is partly a function of familiarity, with more familiar odors being redolent of fewer smells than unfamiliar odors (Mingo and Stevenson, 2007). How many odors a smell reminds one of is not related to the chemical complexity of that odorant, as redolence appears to be a psychological construct and a reliable one at that even for odors that remind someone of many smells (Dravnieks, 1982). Redolence judgments normally occur when we are asked to describe an odor, and so perhaps they normally follow perception rather than accompany it (e.g., sniff then rate/describe). If the content of consciousness is generally unitary (or perhaps, at a maximum, ternary), and if this is followed by redolence judgments where the odor may remind us of many other odors (i.e., more than three), then for olfaction what is accessible (*perhaps* redolence judgments) at least equals and probably exceeds conscious content.

Is it possible then to explain this seemingly unusual “content of consciousness” with reference to the functional issues raised in Section The Human Olfactory System? There does not seem to be any obvious proximal function served by this form of “content of consciousness.” Distal functional explanations may be more promising, as limited content could be a product of the relatively restricted neocortical processing resources devoted to olfaction (Kaas, 2013). One way of instantiating this at the psychological level of explanation would be to make odor selection (i.e., the content of consciousness) an automatic procedure. A potential implication of this would be that there is no phenomenal/access distinction for olfaction, instead all we have is automatically mandated access consciousness. In vision and audition a combination of exogenous and endogenous attentional processes dictate what object or objects are selected for further processing, and certain aspects of this are under volitional control (Sperling, 1960; Van Rullen and Koch, 2003). Some limited volitional control may also be evident in olfaction, notably during the search for a particular odor (Zelano et al., 2011). However, this may differ substantially from the major senses because for olfaction “searching” may only work effectively if it happens to coincide with the olfactory systems built-in tendency to focus on chemicals that are *new* to the search environment. Searching for a habituated odor may be ineffective, making the use of a conscious strategy more limited in this sense.

The advantage of thinking about the content of olfactory consciousness as being the result of an access only system, is that it readily accounts for why its information content (in terms of the number of objects which can become the focus of attention) is relatively low when contrasted with the apparent phenomenal richness of the major senses. It certainly does not have to be this way as the brain undoubtedly processes a considerable amount of information about the individual chemical components of an odor, and this could go to make a rich phenomenal experience, perhaps as it does in the major senses. However, for olfaction this information does not seem to be consciously accessible (e.g., Gottfried et al., 2006).

#### The primacy of affect

Engen noted that “Functionally, smell may be to emotion what sight or hearing are to cognition” (Engen, 1982, p. 3). It should not be surprising then given Engen's quote that an important aspect of olfactory experience is the hedonic tone that accompanies smelling. Some researchers have even argued that the affective response to a smell actually reflects the primary response, being more important than perceptually based means of recognition (Yeshurun and Sobel, 2010). While this strong claim may be unlikely, partly because recent experimental work indicates that recognition-related processes occur before hedonic judgments (Olofsson et al., 2012), there is good support for the idea that affect is a more central part of the olfactory experience than it is for vision or audition. One important line of evidence has come from multidimensional scaling experiments, which can be used to determine the underlying dimensions that mediate similarity judgments. A consistent finding in this literature has been that the primary dimension is typically hedonic (e.g., Schiffman, 1974). Another line of evidence comes from examining olfactory memories, which have been found to be more emotionally evocative than memories retrieved by comparable visual or auditory cues (e.g., Herz, 2004). Finally, an analysis of olfactory related words reveals them to be on average more affect laden, with unpleasant terms outnumbering pleasant terms, relative to words associated with vision or audition (Ehrlichman and Bastone, 1992).

While affect seems to be a central part of the olfactory experience, it has been noted that if people are asked to provide a list of their most affect-laden experiences, smells will not generally figure high on this list (Ehrlichman and Bastone, 1992). Rather visual and auditory experiences will tend to dominate in seeming contradiction to the arguments presented above. What seems to be special about olfaction is that the object that causes the smell seems to actively *contact* the body, that is it seems to be phenomenologically more proximal than vision or audition (e.g., Rouby and Bensafi, 2002). So the hedonics for smell feels more direct and visceral than the hedonics associated with vision and audition. This is best illustrated by the emotion of disgust. This emotion is frequently triggered by smell, probably because volatile chemicals are often a good cue for disease-related objects and events (Oaten et al., 2009). A characteristic feature of disgust is that contact with an elicitor of this emotion *feels* contaminating (e.g., Sherman et al., 2012), thereby compelling movement away from the object (Rozin and Fallon, 1987). While we might dislike looking at fake dog feces or plastic vomit, synthetic fecal or vomit odors still compel avoidance even if we know they are not real. Such smells just *feel* bad.

Functionally, it has been presumed that the primacy of affect reflects the need for rapid withdrawal or approach, without the need for (presumably) longer cognitive appraisal (Yeshurun and Sobel, 2010). Perhaps there is some merit in this idea when it is applied in the context of ingestive behavior, where detection of microbial contaminants or natural poisons, may require rapid rejection of a food from the mouth before it is swallowed. However, this particular set of circumstances would be rare, because if an off-smell were detectable in the mouth, it would almost certainly be detectable by the nose prior to ingestion. More generally, we seem well able to avoid dangerous situations when they are revealed to us by vision or audition (e.g., a looming object, avoiding road traffic, fire alarms etc.), suggesting that negative affect is not a necessary prerequisite for rapid withdrawal. That people can effectively avoid dangerous situations using non-affective means implies that affect-based processing is not uniquely effective in this regard. It could then be that the affect that routinely accompanies olfactory experience is actually just the product of economical cognition resulting from limited neocortical resources (i.e., no or limited relative to the other senses, dedicated unimodal neocortical tissue). Thus, this position contrasts with the idea that affect generation confers some special advantage over cognition in terms of promoting faster or more effective avoidance (although the bad contamination “feel” of olfactory disgust elicitors may be something special).

#### Universal synesthesia

While it has been known for many years that people routinely describe certain food odors as *smelling* of particular tastes (e.g., Harper et al., 1968), the unusual nature of these observations only started to attract attention relatively recently (e.g., Frank and Byram, 1988). Importantly, *pure* tastants do not trigger smell sensations (noting that tastants are often contaminated by volatile chemicals; Mojet et al., 2005), it is only smell sensations that can seem to trigger *both* smell and taste sensations even if they have no contact with taste receptors (e.g., Sakai et al., 2001). A further issue is that taste is a discrete sensory system from smell. Taste receptors are located mainly on the tongue and they send information to the brain via a different route to that of smell (Schiffman, 2002). Smell and taste information first converge in the brain, in secondary neocortical structures (Rolls, 1999). To say then that something smells “sweet, sour, bitter, salty or meaty” is directly akin to saying that visual objects routinely trigger sound sensations and vice versa (e.g., a telephone looks ringing).

While synesthesia has typically been explored in the context of the relatively rare individuals with grapheme-color synesthesia (e.g., Mroczko et al., 2009) or some other variant (e.g., lexical-gustatory synesthesia; Ward et al., 2005), a notable aspect of odor-induced tastes is that they seem to be experienced by everyone (Stevenson and Tomiczek, 2007). Although there are similarities between these rare synesthesias and odor-induced tastes, especially in the stability of these experiences over time, their automaticity and involuntariness, there are also differences. The most important seems to be the largely idiosyncratic nature of many synesthetic inducer-concurrent mappings (i.e., why *should* the letter A induce red color?; Deroy and Spence, 2013).

There are several possible mechanisms that could account for odor-induced tastes. One obvious one is that the use of taste-based terms to describe food odors could be metaphorical (e.g., people are sometimes described using taste-based terms). This appears unlikely. The most obvious metaphor is for affect as with saying someone is sweet. Several studies have now shown that odor-induced taste experiences are dissociable from odor hedonics (e.g., Yeomans and Mobini, 2006). A further and related alternative is that people simply employ taste terms for odors that they know explicitly are associated with particular tastes—in other words it is a purely verbal/semantic association (smell-knowledge). Several pieces of evidence speak against it being a verbal/semantic phenomenon: (1) odor-taste associations can be acquired implicitly, in the absence of explicit verbalisable knowledge of the odor-taste pairings (e.g., Stevenson et al., 1998; Brunstrom, 2004); (2) odor induced taste characteristics are reliably present even when participants are unable to identify the odor in question (e.g., Stevenson et al., 2012); (3) odors that induce particular taste sensations have physiological effects that parallel those observed when experiencing an actual gustatory experience (Prescott and Wilkie, 2007); and (4) a growing body of evidence indicates that odors can induce taste experiences in animals, and that these are acquired in the same way as in humans (e.g., Harris and Thein, 2005; Gautam and Verhagen, 2010). While metaphorical or verbal-semantic mediation accounts cannot be wholly excluded as explanations of odor-induced tastes, they seem unlikely.

A further explanation is that certain odors can activate brain regions also active during tasting, resulting in a taste-like experience that is highly perceptually similar to the experience induced by tastants on the tongue (e.g., the sweetness of sucrose on the tongue). That is odor-induced tastes arise from odor-taste associations that are based upon a link between these two percepts, such that the odor percept comes to activate the taste percept. This conclusion has emerged from nearly 20 years of research. Key findings include the observation that: (1) odors that smell of a particular taste can enhance the intensity of that tastant when they are added to it (e.g., Frank and Byram, 1988); (2) odors acquire taste-like properties via associative learning during flavor perception (e.g., Stevenson et al., 1998); (3) odors that smell of a particular taste facilitate identification of that taste (White and Prescott, 2007); (4) tastants that taste the same as an odor smells, facilitate the detection of that smell (e.g., Dalton et al., 2000); and (5) patients with centrally based taste impairments also have selective impairments in perceiving odor-induced tastes (e.g., Stevenson et al., 2008). Together with many other supportive findings not summarized here (see, Stevenson, 2012), these findings suggest that odors can induce taste-like sensations.

It has been suggested that the function of odor-induced taste is to assist in identifying prospective foods, so as to aid prediction of their likely taste in the mouth (Stevenson and Tomiczek, 2007). Currently, and as in our evolutionary past, human food selection is heavily dependent upon color vision, as it is in our fruit-eating primate ancestors where it evolved (Regan et al., 2001). Detecting a food's likely taste via smell is an adaptation that may have had much greater functional significance for animals less reliant on vision (although as in humans it may well augment visual decision making; Hiramatsu et al., 2009), especially rodents. Indeed, rodents can perceive “tasty-smells” just as we can (e.g., Gautam and Verhagen, 2010) and so our ability in this regard may represent the conservation of a function, which no longer confers a major benefit (beyond, perhaps, providing an insight into what it might be like to experience smell like a rat; Nagel, 1974).

#### Conclusion to content of consciousness

Olfactory conscious experience appears to be mainly singular with one odor event perceived at a time. The large array of objects potentially available to visual attention during perception contrasts with the more limited range available to smell. An odor will be redolent of other odors, it will be affectively toned, and if perceived before in a food, it will probably have taste-like qualities. The olfactory percept seems to directly encapsulate its meaning (especially taste, affect), and it does so with minimal effort, notwithstanding the making of redolence ratings. While visual and auditory percepts also contain considerable inherent information (e.g., depth, location, color, etc) they do not normally contain sense experience drawn from another modality (a quantitative difference). In addition, visual and auditory percepts are not usually accompanied by the visceral feel of affective contact (a more qualitative difference).

It might in theory be possible to explain these differences by reference to the demands and constraints of the olfactory system. However, there does not seem any compelling connection between the demands and constraints identified in Section The human olfactory system, and the unusual characteristics of human olfactory conscious content. Instead, we suggest that most of the differences in conscious content may be explained by reference to olfaction's limited neocortical processing resources, the exception being odor-induced tastes, which may be a vestige (but still useful) of a once more adaptive food selection system. In the main, the processing differences examined in this section may reflect particularly economical forms of perception, cognition, and consciousness.

### Attentional processing

#### Mouth and nose

Odorants access the olfactory receptors either orthonasally via the nostrils on the face, or retronasally via the internal nostrils at the back of the throat during eating and drinking. One important distinction that has emerged here is that between content and modality awareness (Rozin, 1982; Stevenson, 2013b). When an odor is smelled at the nose, a person knows both that an odorant is present and that the sensory system involved is smell. When an odor is sensed in the mouth as part of a food (retronasally), alongside the anatomically discrete senses of taste and somatosensation (mouth feel), the person is capable of perceiving retronasal odor quality, intensity and hedonic properties. However, this is not routinely accompanied by an awareness of the sense modality (olfaction) involved in its perception (Stevenson, 2009a). While this may apply to naïve participants (as for most of the discussion below), it is likely that experts do have an awareness of olfaction's role in flavor, but this topic has not been well explored. For naïve participants then, sense experience in the mouth is routinely described as flavor or more colloquially as “taste,” but not as smell (note that “taste” in single quotes refers here to the colloquial term for flavor, while taste refers to gustation).

The evidence for a dissociation of content and modality awareness in the mouth is quite strong, although it has not received a lot of contemporary research attention. Rozin (1982) asked participants which term they would use to describe a range of different foodstuffs, including several that had significant olfactory components. He found that the terms “taste” and flavor were used interchangeably, although flavor tended to be used more when the item had a greater olfactory component. Rozin (1982) also observed that of all of the major language groups that he had searched, none, including English, had a word that meant “smell in the mouth” (note, however, that experts clearly do have a term for this distinction—retronasal olfaction—although its meaning would probably be unknown to most naïve participants). Another manifestation of the content modality dissociation is seen in people who have lost their sense of smell. When people first present with this problem, they frequently complain of experiencing both a loss smell *and “taste”* (Deems et al., 1991). Upon examination it is evident that taste is intact, rather food now “*tastes”* bland as a consequence of the loss of smell.

While participants may attribute the portion of their sensory experience of food that rightly belongs to olfaction, to “taste,” the olfactory content of that experience is clearly perceived (Small et al., 2005). The olfactory component of food is a major factor in our enjoyment of eating and drinking, as is made evident when one has a cold and retronasal olfaction becomes impaired. More formal psychophysical tests of peoples ability to identify particular odors in the mouth reveal a capacity to do so that is similar but somewhat poorer than the capacity at the nose (e.g., Marshall et al., 2006). The food industry clearly believes that people can experience smells in their mouth. They spend large sums of money on sensory evaluation panels that reliably judge many purely olfactory attributes of food, which result from the release of volatiles during eating and drinking (Moskowitz and Hartmann, 2008).

One way to consider these findings is to regard taste and smell in the mouth as one perceptual system—taste (but one could equally use the term flavor)—as originally suggested by James Gibson (1966). Gibson's idea can be operationalized by considering taste and smell as having a shared attentional channel in the mouth (Stevenson, 2013b). Although this dual-channel account has not been well investigated, it is consistent with two important findings. First, it does not seem possible to attend to a smell in the mouth without also attending to a co-present taste and vice versa, suggesting that both these senses are entwined (e.g., Ashkenazi and Marks, 2004). Second, if an odor is sniffed at the nose and a taste is placed in the mouth, the presence of the taste can generate an illusory transfer of the location of the odor from nose to mouth (Stevenson et al., 2011). That is the orthonasal smell now appears to be part of a flavor in the mouth. This phenomenon does not occur if an odor is sniffed at the nose and a tasteless somatosensory stimulus is placed in the mouth instead (e.g., a viscous fluid). Together, these findings suggest a special connection between taste and smell that is not shared between smell and oral somatosensation.

Functionally, a case can be made for the idea that the brain needs to connect olfactory information arising in the mouth with olfactory events in the environment so as to aid smell-based food selection (now or at least in the past). Irrespective of whether this view is correct, it still leaves the problem as to the benefit, if any, served by not knowing that smells in the mouth are smells. We suggest two possibilities. One is that it may be more efficient to learn the relationship between a food and its immediate and delayed consequences, if this information is automatically associated (i.e., within an attentional channel, rather than associations between channels). One consequence of this may be evidence of learning even in the face of contradictory explicit knowledge (e.g., falsely associating the nausea from cancer chemotherapy with a food, but knowing the food was not responsible; Bernstein, 1985). A second possibility is that there may have been no evolutionary pressure for awareness of smells in the mouth, and so we just retain an information processing system that predates a conscious reflective component, which is usually deemed necessary for human associative learning (e.g., Shanks, 2010).

#### Re-attending to smell

In many respects, olfaction shares with vision and audition basic aspects of attentional processing (Keller, 2011). Strong, unpleasant or novel odors may involuntarily attract our attention, and we can selectively attend to the olfactory modality, enhancing our reaction time to events in this channel (e.g., Spence et al., 2001). One reason to suspect that attentional processing differences do exist comes from the unusual neural architecture of olfaction (Smythies, 1997). Unlike the major senses, which route all incoming information via the thalamus, the olfactory system is unique in having two routes to neocortex, a thalamic relay and a direct link (Tham et al., 2009). Thus, the olfactory system may be able to transmit information to the neocortex independently of the thalamus. This is important because the thalamus has been presumed to play a key role in attentional processing in the major senses (e.g., Portas et al., 1998).

Recent work has suggested that at least one particular aspect of attentional processing may be different for smell. As described earlier, the olfactory primary cortex undergoes a rapid reduction in neural response to continued chemical stimulation (e.g., Sobel et al., 2000). The presumed reason for this is so that the system is ready and able to detect new odorants as they arise. Importantly, this process of adjustment is principally a cortical change (or more properly a paleocortical one), and not a loss of sensitivity at the receptor level. In fact animal work shows convincingly that olfactory receptors retain sensitivity to an odorant that no longer generates any neural response in primary olfactory cortex (Wilson and Linster, 2008). The cortical locus of this reduced responsivity, combined with retained receptor sensitivity, suggests that it may be termed habituation (i.e., a brain-based phenomenon) rather than sensory adaptation (i.e., a receptor-based phenomenon), a division long recognized in the literature (Thompson and Spencer, 1966). In the major senses it is relatively easy to voluntarily attend to stimuli that are habituated. In the classic example of the ticking clock, one can voluntarily attend to the sound, but as attention is drawn to other stimuli the ticking again appears to pass out of consciousness (James, 1890).

This does not seem to be the case for the olfactory system as an experiment recently conducted in our laboratory suggests (Mahmut and Stevenson, submitted). Participants were placed in an odorized room and asked to describe its smell using redolence and certainty ratings. One group was then continuously exposed to the smell, but only in one nostril (this being counterbalanced across participants), the other nostril being blocked (recall that each side of the nose has its own discrete olfactory epithelium). Performance, in this group of subjects of their open nostril reflects the effects of peripheral adaptation *and* central habituation, while performance in their blocked nostril just reflects central habituation, which is bilateral (Cain, 1977). The other group of participants had both nostrils blocked, so as to equate exposure created when participants removed the nose plugs to make ratings of the room's odor. After a period of around 20 min exposure, we asked both groups to again describe the room's smell using redolence and certainty ratings. The key result is in the group that had just one nostril blocked, with the other open throughout exposure. When we tested the nostril that had been blocked throughout exposure, they were unable to describe the room's smell when their attention was directed toward it, relative to the way they had at the start of the experiment. This reflects the effect of centrally based habituation, as this nostril (and its associated receptors) had minimal prior exposure to the odor, and so no sensory adaptation should have occurred. Participants in the other group, who previously had both nostrils blocked, were still able to describe the odor in the same way as they had at the start of the experiment (i.e., no receptor adaptation or habituation). In sum, participants asked to attend to a centrally habituated odor seemed unable to voluntarily recover its conscious representation.

As we noted earlier, the olfactory system has to detect new odorants against the background of currently present odorants, and habituation may play a significant role in this process (noting that the persistence of one odorant will not necessarily block perception of another). While it would be tempting to describe failure to re-attend to a habituated odor as a consequence of keeping the olfactory system optimized to detect the advent of new odorants, this explanation seems inadequate. This is because all of the major senses face a similar problem of constant stimulation against which new events have to be detected, and all of them also show habituation (Thompson and Spencer, 1966). However, re-attending to habituated stimuli is still possible in the major senses (James, 1890). Perhaps then it is the nature of the chemical stimulus, which somehow precludes our ability to re-attend to a habituated odor, but it is not obvious why this would be so either. An alternative perspective based again on limited neocortical processing resources (see Section Mouth and Nose) may be needed. It is, we suggest, the absence of dedicated neocortical processing that prevents us from re-experiencing a habituated smell.

#### Conclusion to attentional processing

The division of olfaction into a sense of smell at the nose, where we are aware of both the modality and content, and a sense of “taste or flavor,” where we are aware of content, but not modality, is probably an attentional phenomenon (Stevenson, 2013b). We suggested this might arise because either it is a more efficient means of learning or because it may be that this form of information processing has simply remained conserved over evolutionary history. The second attentional processes examined in this section concerned attending to a habituated odor. This can be conceptualized, as with our discussion of perceiving one smell at a time (Section Mouth and Nose), as being a further consequence of automatic stimulus selection, which in turn may result from limited neocortical processing resources.

### Post-perceptual processing

In the visual and auditory domains it is generally accepted that: (1) people can rehearse sounds or images in domain-specific working memory modules (respectively the visuo-spatial scratch pad and the articulatory loop); and (2) that these short-term memory processes are intimately connected with the capacity for conscious visual and auditory imagery (Baddeley and Andrade, 2000; Postma and Barsalou, 2009). The olfactory literature on these topics presents a far more complex and seemingly confusing picture. First, after decades of disagreement, it now appears that *trying* to imagine an odor does have several detectable consequences: (1) it activates brain regions, including primary processing areas, which are also active during real smelling (e.g., Djordjevic et al., 2005); (2) it improves various psychological capacities, such as enhancing the detection of threshold level odors (Djordjevic et al., 2004) and priming (Tomiczek and Stevenson, 2009); (3) it mimics various psychophysical parameters, relating to olfactory interactions, intensity and quality (e.g., Carrasco and Ridout, 1993); and (4) it generates olfactomotor responses (sniffing behavior) that closely approximate what is observed during actual smelling (e.g., Bensafi et al., 2003). While, a number of studies have failed to find improved psychological capacities (e.g., Crowder and Schab, 1995) or mimicking of psychological effects observed with real odors (e.g., Herz, 2000), the weight of evidence suggests that trying to imagine a smell can influence a variety of psychological, physiological and neural variables, in much the same way as actual smelling can. However, what is at issue here is whether these various effects, which result from trying to imagine an odor, are mediated or accompanied by a conscious representation of the imagined smell. We suggest they are not.

There are several reasons for thinking that trying to form a conscious odor image, or for that matter attempting to rehearse an odor representation in some form of “mind's nose,” may not normally occur. The most obvious reason for doubting that it does comes from simply asking people what they experience when they try to form mental images in different modalities. In all of the studies that we are aware of olfaction is either reported as the modality in which it is most difficult to imagine a perceptual event (e.g., Betts, 1909; Ashton and White, 1980) or it is the modality where participants most frequently report being unable to form any sort of conscious image (e.g., Brower, 1947; Lawless, 1997). These findings do not seem to reflect a broader failure to be *able* to notice olfactory experiences in the absence of appropriate stimulation. Indeed, there is a large literature documenting reports of olfactory hallucinations in people who are not psychotic, but who experience epilepsy, brain tumors and migraine for example (see Stevenson and Case, 2005; Stevenson and Langdon, 2012). So when people say they cannot experience an odor image or that it is vague or indistinct, there seems no obvious reason to doubt the validity of their reports.

A further line of evidence concerns the relationship between psychological and psychophysical performance measures obtained during odor imagery experiments, and self-reports of imagery ability. For visual imagery there are well-established links between these two types of variable (e.g., McDermott and Roediger, 1994; Baddeley and Andrade, 2000). In olfaction, the links between the two appear to be weak at best. Djordjevic et al. (2004) failed to find a correlation between self-report ability and performance on their detection task, although this relationship did emerge when tested just in females. In fact Lyman and McDaniel (1990) are the only group to report a significant correlation between imagery and task performance, but this study has been criticized, as it is unclear whether the reported imagery performance was mediated by verbal codes (see Stevenson and Case, 2005). Other studies have failed to find any link with performance, including Lyman (1988), and Tomiczek and Stevenson (2009). The latter study explored in some detail the predictors of enhanced imagery performance. Participants reported ability to consciously experience an odor image was not found to be a predictor in any of their three experiments. We suggest based on these findings that while there may be good evidence that attempting to imagine an odor can generate a number of effects that broadly parallel real smelling, the evidence that these are accompanied by a conscious image is weak at best. This does not seem to be the case in the major senses, and it is possible that it may not be the case in olfactory experts either, although the evidence basis for this assertion is currently too small to be definitive (see Royet et al., 2013).

A similar picture also emerges in the olfactory short-term memory literature. Yet again, there is good evidence that there is a capacity for short-term memory in olfaction (e.g., White, 1998; Andrade and Donaldson, 2007). What is not clearly established is the representational code that underpins this, and whether it is instantiated discretely (i.e., a short-term olfactory module) or as a component of long-term memory (e.g., Yeshurun et al., 2008; Johnson and Miles, 2009). Evidence that one can hold a conscious representation of an odor in short-term memory once the stimulus had been removed, and perhaps even rehearse this image, is scarce. One potential line of evidence is the presence of primacy effects in the serial position curve, but these have not generally been found for olfactory stimuli (e.g., Miles and Hodder, 2005). Another concerns the two-back task (i.e., is the current stimulus the same as the one smelt before the last one?), which may require some form of active rehearsal to maintain and update working memory. Although olfactory performance on this task seems to depend heavily on participants naming the odor, there is evidence to suggest that the two-back task can be performed even when the odor is unfamiliar and thus likely to be difficult to name (Jonsson et al., 2011). There is then as yet little evidence that odors can be consciously rehearsed in some form of olfactory short-term store.

Based upon current evidence, it looks as if there might be a dissociation between an operational capacity for short-term storage, imagery and rehearsal, and an associated conscious state. That is, these cognitive operations do not seem to be routinely accompanied by a conscious representation. One possible functional benefit of such a conscious*-less* cognition is that it precludes troublesome interference between detection of odors new to that environment and any on-going cognitive operation. However, this appears a weak argument. First, in general, people do not seem to try and remember odorants, imagine them or whatever. While of course this may be because they cannot do so, there would not appear to be much day-to-day call for most of us to try and do so. Second, the major senses seem able to manage imagery-reality confusions, except where these are deliberately engineered to confuse participants by making the real stimulus weak (e.g., Segal and Fusella, 1971; Mathews et al., 2013). Of course it could be that because olfactory percepts are somewhat less vivid than the other senses (e.g., Cain and Algom, 1997), this has prevented the development of imagery-related processing (i.e., because if an imagery capacity evolved it led to fatal confusions between imagination and reality). If this were correct, then this in turn would raise the question as to why olfactory percepts are less vivid or weak. Answering this question would probably lead to the same conclusion as the one that prompted this discussion (i.e., absent conscious processing of imagery and rehearsal). That is olfaction seems to have this feature not because it faces a unique set of challenges, but because it has access to only limited neocortical resources. These limited resources produce tangible effects, one of which may be cognition with minimal conscious representation.

## Discussion

The main idea explored in this manuscript is that there are function-related reasons for the way in which consciousness and cognition are configured in human olfaction. In an earlier examination of olfaction's unusual psychological features, Stevenson (2009b) implied that proximal functional factors might be responsible, but he did not explore this issue in any depth. In the current article, which addresses this more directly, it would seem that many of the problems that the olfactory system has to solve to meet its basic function (e.g., recognizing biologically significant chemical mixtures) are in fact *common* to all of the senses. Even a relatively unique problem, such as the persistence of chemical stimuli in the environment, should not unduly constrain cognition. For example, the somatosensory system faces a similar problem of stimulus persistence (e.g., clothes), but this does not seem to preclude turning attention back to the way, for example, of how ones clothes feel. This does not seem to be the case for olfaction. Before turning to the more general explanation advanced here, it is important to note that at least one class of proximal function, unique to olfaction, does seem to have explanatory power. This is the need to link the immediate and delayed consequences of ingestion with the smell of food. This may have contributed to three unusual aspects of olfactory cognition, namely the primacy of affect, odor-induced tastes, and the lack of modality awareness for odors in the mouth. Notwithstanding, even these function-related features may be of lesser current value since the advent of color vision and the allocation of neocortical resources to this sense in our primate ancestors.

The main argument to emerge from this review is that many of olfaction's unusual features may be attributed to its limited allocation of neocortical resources. The capacities olfaction does have result then from its primary processing by many limbic system structures, with its paleocortical and subcortical centers. We have further suggested that the failure of olfaction to take space in the burgeoning neocortex of primates and early hominoids may have come about because chemicals represent a poor medium for flexible communication. The rapid expansion of neocortical tissue in our human ancestors left olfaction languishing as a minor sense (Kaas, 2013), without the need for the neocortical resources necessary to support the manipulation of units of sensory meaning, and their formation into ideas to communicate within the brain and between people. Importantly, this is not to say that olfaction is incapable of transmitting information. Olfaction represents information affectively, and can trigger powerful emotional states (e.g., disgust), and this can be communicated within the brain and to others (e.g., via facial expression). Nonetheless, this communicative capacity is considerably less flexible than one where perception, semantic memory and verbal thought are highly interconnected, as they are for all of the major senses (Revonsuo, 1999). One place this can be seen clearly is in the very well documented problem that most people have in naming even common odorants in the absence of visual or auditory cues (e.g., Cain et al., 1998). Another is the limited access they have to semantic memory systems in the absence of a name (e.g., Stevenson and Mahmut, 2013b). There is no doubt that olfaction is an effective sensory system, but it is a highly limited one relative to all of the other neocortical dependent perceptual systems that we possess.

The claim of limited neocortical resources is not as un-testable as it may at first seem. In a novel line of work, Plailly et al. (2012) have been exploring how olfactory perceptual expertise induces various types of functional reorganization of the brain. It may be that extensive practice can produce increases in neocortical processing power for smell, sufficient to propel what may be unconscious processes in naïve participants into conscious ones for experts. This is certainly what the experts' claim (e.g., Gilbert et al., 1998) and interestingly this seems to be accompanied by the creative use of these cognitive operations to imagine new perfumes or flavors and communicate these ideas to other professionals.

This leads to the second idea we wanted to explore in this manuscript, namely how cross-modal comparisons can be valuable in pointing to the functional benefits that accrue from conscious processing. First, we suggest that the fact that many successful olfactory operations can seemingly occur without conscious awareness, while being potentially conscious in the major senses, seems to imply that consciousness has a function (i.e., if it has not, why not stick with an olfaction-like conscious-*less* information processing system?). Second, we suggest that one benefit of conscious processing is the availability of this information for further manipulation, typically for creative and communicative ends. Not surprisingly, it is with this end in mind that the long training period that accompanies olfactory expertise is aimed, and the ability to *control* what information is combined or contrasted with other information seems to be a hallmark of conscious processing in vision and audition, and one that is typically lacking in olfaction—except perhaps in experts.

To us, the most striking differences between olfaction and the major senses is in the content of consciousness itself. Here olfaction has far more limited content than the major senses. One argument we make is to suggest that the content of olfactory consciousness may be limited because all we can experience is the access component. On this basis we do not have phenomenal olfactory consciousness, which is perhaps a consequence of information processing in paleocortical tissue. Perhaps then paleocortical tissue cannot support conscious representations, and while there is evidence that could be mustered favoring this possibility [e.g., notably the temporal aspects of conscious content when smelling seem more correlated with secondary olfactory cortex (orbitofrontal neocortex) than they do with primary olfactory paleocortex (the piriform cortex)], it again points to the interesting possibilities that can emerge when contrasting the senses. Finally, there are now many theories that claim to explain different aspects of conscious processing. While it is beyond the scope of this manuscript to evaluate them all with respect to olfaction, we want to end by pointing out how valuable this might be. Here, we have focused on the phenomenal/access distinction. However one chooses to interpret the data mustered in this article, they do suggest that the phenomenal/access distinction is not the same for olfaction as it is for the major senses. Examining other theories may be equally revealing.

## Author contributions

Richard J. Stevenson and Tuki Attuquayefio jointly prepared and wrote the manuscript.

### Conflict of interest statement

The authors declare that the research was conducted in the absence of any commercial or financial relationships that could be construed as a potential conflict of interest.
